# Promoting ‘testicular awareness’: Co‐design of an inclusive campaign using the World Café Methodology

**DOI:** 10.1111/hex.13898

**Published:** 2023-10-25

**Authors:** Mohamad M. Saab, Varsha N. Shetty, Megan McCarthy, Martin P. Davoren, Angela Flynn, Ann Kirby, Steve Robertson, Gillian W. Shorter, David Murphy, Michael J. Rovito, Frances Shiely, Josephine Hegarty

**Affiliations:** ^1^ Catherine McAuley School of Nursing and Midwifery University College Cork Cork Ireland; ^2^ Sexual Health Centre Cork Ireland; ^3^ School of Public Health University College Cork Cork Ireland; ^4^ Department of Economics, Cork University Business School University College Cork Cork Ireland; ^5^ School of Allied Health Professions, Nursing & Midwifery, Faculty of Health University of Sheffield Sheffield UK; ^6^ Drug and Alcohol Research Network Queen's University Belfast Belfast UK; ^7^ School of Computer Science & Information Technology University College Cork Cork Ireland; ^8^ Department of Health Sciences, College of Health Professions and Sciences University of Central Florida Orlando Florida USA; ^9^ HRB Clinical Research Facility University College Cork Cork Ireland

**Keywords:** health promotion, men's health, qualitative research, sexual and gender minorities, testicular diseases, World Café

## Abstract

**Introduction:**

Testicular cancer is the most common cancer in men aged 15–44 years in many countries. Most men with testicular cancer present with a lump. Testicular symptoms are more likely to occur secondary to benign diseases like epididymo‐orchitis, a common sexually transmitted infection. Gender and sexual minorities are at an increased risk of testicular diseases and health disparities. The aim of this study was to co‐design an inclusive community‐based campaign to promote testicular awareness.

**Methods:**

This study uses the World Café methodology. Participation was sought from Lesbian, Gay, Bisexual, Transgender and Queer+ friendly organisations, testicular cancer survivors, health policy makers, media and marketing experts and graphic designers. Participants engaged in three rounds of conversations to co‐design the campaign. Data were collected using drawing sheets, artefact cards, sticky notes, coloured markers and a voice recorder. Deductive thematic analysis was conducted.

**Results:**

Seventeen individuals participated in the study. Six themes emerged from the analysis as follows: (i) online communication; (ii) offline communication; (iii) behavioural targeting and education; (iv) campaign frequency and reach; (v) demographic segmentation; and (vi) campaign identity. The use of social media for campaign delivery featured strongly in all conversations. Participants also recommended offline communication using posters and radio/television advertisements to scale up the campaign and achieve wider reach. Advertisements to overcome embarrassment surrounding testicular health were particularly recommended. Participants emphasised that campaign delivery must be dynamic whilst ensuring that the health‐promoting messages are not diluted or lost. They stressed the importance of being inclusive and tailoring the campaign to different age groups, gender identities and sexual orientations.

**Conclusions:**

Study recommendations will be used to design and deliver the campaign. Future research will be needed to evaluate the feasibility, acceptability, cost and effect of the campaign on promoting testicular awareness and early detection of testicular diseases.

**Patient or Public Contribution:**

A participatory research approach was used to co‐design the campaign with members of Lesbian, Gay, Bisexual, Transgender and Queer+ (LGBTQ+) friendly organisations, LGBTQ+ student bodies, LGBTQ+ staff networks, LGBTQ+ sports clubs, men's health organisations, testicular cancer survivors, health policy makers, media and marketing experts and graphic designers.

## 1 INTRODUCTION

‘Testicular awareness’ is an all‐encompassing concept aimed at familiarising individuals with an intimate part of their body that is seldom discussed, enabling them to detect abnormalities and seek timely medical help accordingly, regardless of the ultimate diagnosis.^1^ ‘The key attributes of “testicular awareness” which facilitate symptom perception and interpretation are as follows: (i) familiarity with own testes; (ii) knowing what is normal versus abnormal; (iii) ability to detect an abnormality; and (iv) knowing own risk factors’ (p. e3).^2^

Having testicular awareness is important because testicular cancer is among the most common cancers in males aged 15–44 years, with 74,458 new cases and 9334 deaths globally in 2020.^3^, ^4^ In Ireland, approximately 176 individuals are diagnosed with testicular cancer each year, with 91% of cases under the age of 50 years. The incidence of testicular cancer has more than doubled in Ireland between 1994 and 2015, with an annual percentage increase of 2.4%.^5^ Testicular cancer, when diagnosed early, is curable with radical orchiectomy.^6^ The 5‐year survival rate for testicular cancer is 95% and the 15‐year survival rate for stage I disease is 99%.^7^ This highlights the importance of early detection and diagnosis, which are associated with reduced need for aggressive treatments,^8^ and lower healthcare costs.^9^

The classical symptom of testicular cancer is a palpable lump.^10^ Lumps, however, do not occur exclusively in testicular cancer. In fact, the likelihood of a lump occurring secondary to benign testicular diseases like epididymo‐orchitis is far higher than testicular cancer.^11^ Testicular torsion (twisting of the spermatic cord) is another benign testicular disease that can cause lumpiness and pain in males under the age of 25 years.^12^, ^13^

Minority groups including gender and sexual minorities are at an increased risk of health disparities.^14^ The incidence of epididymo‐orchitis, the most common cause of testicular symptoms, is highest in the gay community and in men who have sex with men,^15^ emphasising the importance of raising testicular awareness among this population.

Fear of a potential cancer diagnosis, unfamiliarity with one's own testes and embarrassment are known barriers to medical help‐seeking for testicular symptoms.^16^, ^17^ This, added to the ongoing debate around the risks and benefits of regular testicular self‐examination,^18^ led to the development of the concept ‘testicular awareness’.

Literature reviews conducted over the past decade have reported on studies that explored and aimed to enhance men's awareness of testicular cancer and self‐examination.^19^, ^20^, ^21^, ^22^ Conversely, very little is known about men's awareness of benign testicular diseases.^11^ We conducted a search in electronic databases and trial registries to update the abovementioned literature reviews.^19^, ^20^, ^21^, ^22^ Five experimental studies published between 2018 and 2023 were reviewed. Interventions like PowerPoint presentations,^23^ online educational brochures,^24^ video‐assisted teaching,^25^ motivational videos^26^ and a virtual reality game^27^ were successful in raising men's awareness of testicular cancer and self‐examination. Of note, none of the studies purposefully included gender and sexual minorities and only one study addressed benign testicular diseases,^27^ further highlighting the need for an inclusive health intervention to promote testicular awareness.

The aim of this study was to co‐design an inclusive community‐based campaign to promote testicular awareness. Our study sought to answer the following question: What are participants' preferences in relation to (i) campaign format(s); (ii) frequency of delivery; (iii) target population(s); (iv) place(s) of delivery; and (v) identity?

## 2 METHODS

### 2.1 Design

The positive role of participatory research and patient and public involvement in developing effective health interventions is receiving increased attention.^28^, ^29^ The World Café (WC) methodology is a participatory research approach and a cooperative learning method.^30^ It is flexible,^31^ and process‐oriented, which can support small and large group conversations with the goal of generating collective insights and cross‐pollinating ideas around questions that matter.^32^ The WC methodology has proven effective in collecting qualitative data by allowing participants to engage in strategic discussions.^33^

This study was guided by the following seven WC design principles: (i) clarifying the context; (ii) creating hospitable space; (iii) exploring questions that matter; (iv) encouraging everyone's contribution; (v) connecting diverse perspectives; (vi) listening together for patterns and insights; and (vii) sharing collective discoveries.^32^ The Standards for Reporting Qualitative Research checklist was used to report this study.^34^

### 2.2 Sample and context

Ethical approval was obtained. Purposive sampling was used to recruit participants from the south of the Republic of Ireland. We also asked participants to discuss the study with friends and colleagues who met the study inclusion criteria. The target population was adults aged 18 years and above representing diverse participant categories. In line with our research question and the fact that the incidence of common testicular diseases like epididymo‐orchitis is highest in the gay community and in men who have sex with men,^15^ we identified key community partners who would be best placed to co‐design and eventually help deliver the campaign. Therefore, we targeted individuals from Lesbian, Gay, Bisexual, Transgender and Queer+ (LGBTQ+) friendly health organisations, student bodies, staff networks, sports clubs and men's health organisations. We also sought participants who were testicular cancer survivors, health policy makers, media and marketing experts and graphic designers. We sought these groups through publicly available e‐mail addresses and word of mouth. A study information sheet was enclosed in the e‐mail invite, explaining the aim of the study and the importance of promoting testicular awareness. Participation was encouraged by informing potential participants that: (i) their anonymity will be maintained during data analysis and dissemination; (ii) no risks are associated with their participation; (iii) parking and travel expenses will be covered; and (iv) a light dinner will be served. Two e‐mail reminders were sent to maximise participation.

Individuals who expressed interest in participating were asked to provide their availability for the workshop, which took place in the evening to accommodate work, study and other life commitments. There is no consensus as to how many participants should be included in WC workshops; however, Löhr et al.^30^ suggest a sample size greater than 12 is regarded as acceptable.

### 2.3 Data collection

The WC workshop was conducted in March 2023 in an accessible venue on a university campus. Participants completed a sociodemographic questionnaire, which captured their age, gender identity, nationality, sexual orientation, current occupation and role in the WC.

To create a comfortable and welcoming atmosphere that fostered open communication, the venue was transformed into a café‐like setting with three large tables, refreshments were served and participants and researchers wore name tags. The WC workshop was led/hosted by M. M. S., a senior academic who has expertise in the subject and WC methodology. Each table had one facilitator (M. P. D., A. F., J. H.) experienced in the WC methodology and one scribe who took notes (V. N. S., M. M. C.).

**Figure 1:**
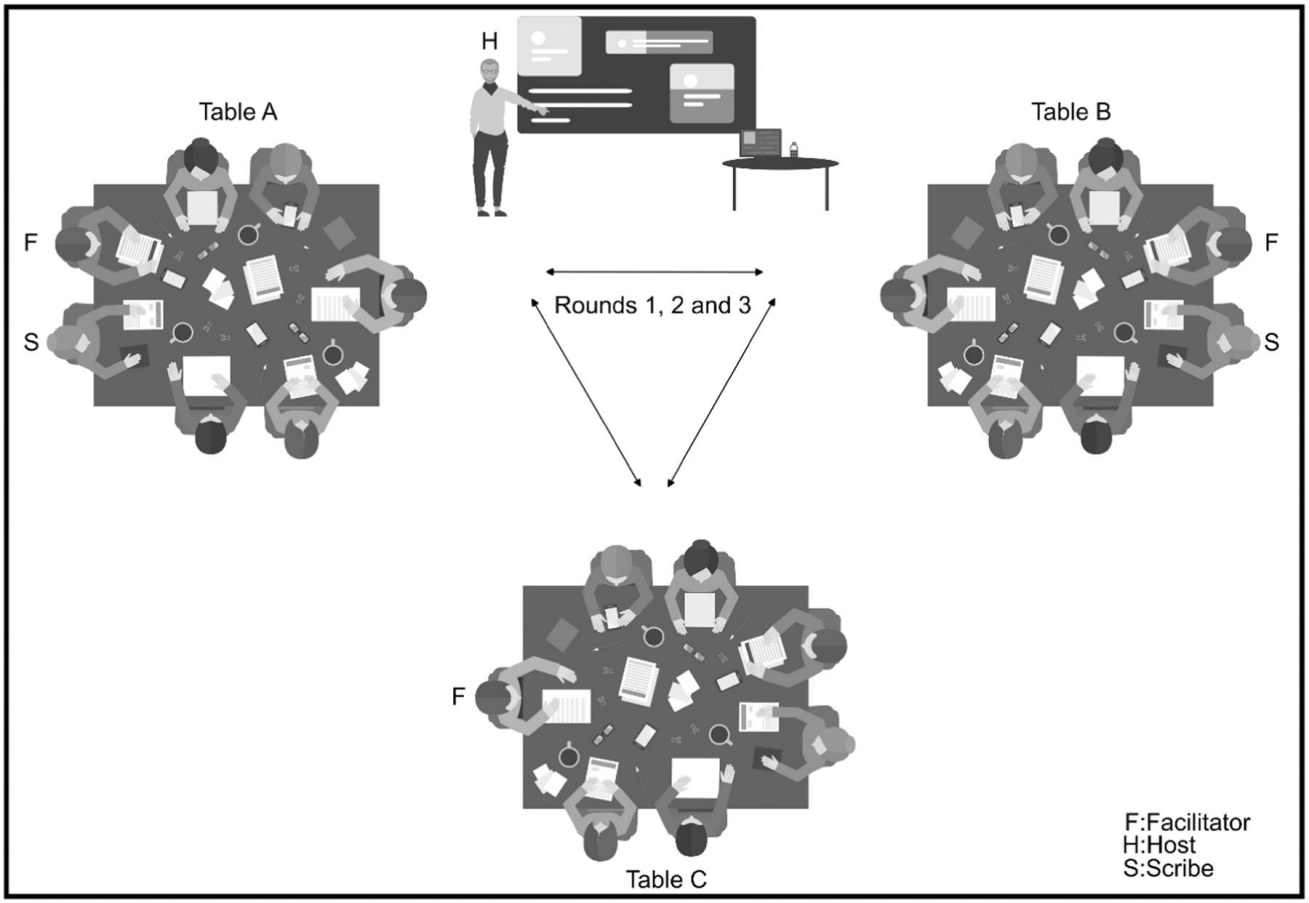
The World Café workshop setting and process used to co‐design the testicular awareness campaign.

**Figure 2:**
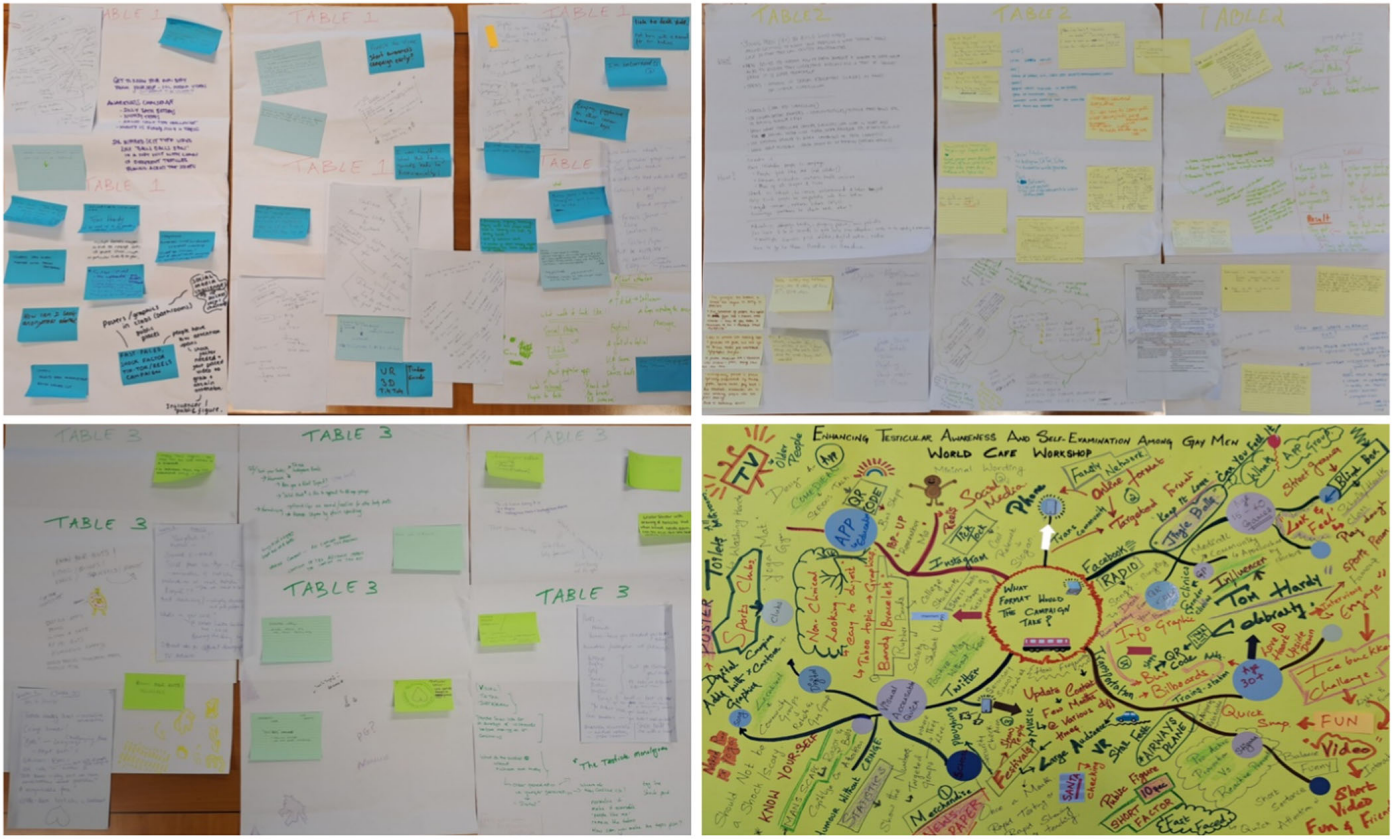
Written artefacts created during the World Café workshop on testicular awareness campaign co‐design.

Participants were randomly assigned to groups of five to six individuals per table. They rotated tables at random for three 20‐min rounds of conversations (Figure 1). Tables were numbered as A, B and C and were colour coded for differentiation. Facilitators used a topic guide to steer the conversations (Table 1). Questions at Table A explored preferred campaign format(s) and delivery, Table B explored the target population and Table C explored campaign identity. Each table had drawing sheets, artefact cards, sticky notes, coloured markers and a voice recorder to record conversations not captured in writing. Artefacts generated during the WC are illustrated in Figure 2.

**Table 1:** World Café workshop topic guide used to guide discussions around the testicular awareness campaign.

Tables	Main questions
Table A	Bearing in mind the three messages that you can see on the screen and the evidence that [the host] shared with you earlier, I would like your opinion on how we can share these messages *(What format would the campaign take? What should it look like?)* How often do we repeat the delivery of the messages in the campaign? *(How frequently should the messages be repeated?)*
Table B	Bearing in mind the three messages that you can see on the screen and the evidence that [the host] shared with you earlier, we want your help with understanding who should be targeted in this campaign? How can we reach out to the target population? Where should the campaign be delivered?
Table C	Bearing in mind the three messages that you can see on the screen and the evidence that [the host] shared with you earlier, how can we build the identity of this campaign? *(Catchphrase, symbol, acronym, slogan, logo…)*

To set the context, the host started the workshop with a 20‐min presentation on: (i) the various testicular diseases and symptoms; (ii) research underpinning the workshop; (iii) the WC methodology; and (iv) the three messages underpinned by the concept ‘testicular awareness’ that the campaign would set out to deliver; that is, promoting awareness of the normal look and feel of one's own testes, what risk factors, signs and symptoms to look for and where to get help for symptoms of concern.^2^ These messages remained on display during the workshop.

The host kept time, introduced facilitators and scribes and engaged participants in an ice‐breaker activity where they shared a random fact about themselves with other participants. Each facilitator then started with a question. Participants were then given 5 min to note down their initial thoughts using any method(s) they prefer. Facilitators moderated discussions and ensured that each participant's voice was heard. Scribes took notes during discussions. After 20 min, participants moved to a different table at random, while facilitators and scribes remained at their assigned tables and welcomed the next group of participants. Facilitators summarised the previous conversations to help build on previous responses and elicit new insights. The same process was used in the three rounds of conversations.

Upon completion of the final round of conversations, a harvesting activity was conducted where each facilitator had 10 min to discuss key findings from their respective table. Participants were also asked to contribute. Findings from harvesting activity were captured using voice recorders and in writing by the host. The workshop lasted 2.5 h.

### 2.4 Data analysis

In WCs, there is no requirement for researchers to follow a classic coding process since relevant insights have already been extracted and discussed during the WC.^33^ However, to tell a coherent and cohesive story, and ensure that all data were captured, deductive thematic analysis was conducted.^35^

Analysing data collected using different methods enhances rigour.^33^ Participants' written responses and facilitators' and scribes' notes were typed and audio recordings were transcribed verbatim to facilitate analysis. A total of 776 codes were generated. After collapsing recurrent codes, a total of 104 unique codes were produced and grouped into subthemes. Themes tying the various subthemes were created. Data analysis was conducted by one researcher (V. N. S.). It was then checked for accuracy and subthemes and themes were finalised in agreement with the host (M. M. S.), facilitators (M. P. D., A. F., J. H.) and scribes (V. N. S., M. M. C.).

## 3 RESULTS

### 3.1 Participant characteristics

Seventeen individuals participated in the WC workshop. The majority were male (*n* = 11, 64.71%), employed (*n* = 14, 82.35%), aged between 18 and 39 years (*n* = 12, 70.58%), Irish (*n* = 14, 82.35%), gay (*n* = 12, 70.59%) and representing an LGBTQ+ friendly organisation (*n* = 12, 70.59%). Participant characteristics are presented in Table 2.

**Table 2:** Characteristics of World Café participants (*n* = 17).

Characteristic	*n*	%
Age (years)		
18–29	6	35.29
30–39	6	35.29
40–49	5	29.41
Gender		
Male	11	64.71
Female	5	29.41
Agender	1	5.88
Nationality		
Irish	14	82.35
Croatian	1	5.88
Spanish	1	5.88
Swedish	1	5.88
Sexual orientation		
Gay	12	70.59
Heterosexual	3	17.65
Bisexual	2	11.76
Employment		
Employed	14	82.35
Student	3	17.65
Current role^a^		
Member of an LGBTQ+ friendly organisation	12	70.59
Member of a sports club	7	41.18
Member of a student body	5	29.41
Healthcare staff	3	17.65
Testicular cancer survivor	2	11.76
Graphic designer	1	5.88
Information technology	1	5.88
Member of a governmental organisation	1	5.88
Member of a men's health organisation	1	5.88

Abbreviation: LGBQT+, Lesbian, Gay, Bisexual, Transgender and Queer+.

^a^ Some participants chose more than one role. *n* corresponds to the number of times the role was chosen.

The following six themes emerged from the analysis: (i) online communication; (ii) offline communication; (iii) behavioural targeting and education; (iv) campaign frequency and reach; (v) demographic segmentation; and (vi) campaign identity. Themes, subthemes and sample codes are presented in Table 3.

**Table 3:** Summary of study findings on testicular awareness campaign co‐design.

Themes	Subthemes	Sample codes
Online communication	Social media	Everyone has a phone/is on social media Facebook^TM^ Instagram^TM^TikTok^TM^ Twitter^TM^
	Videos	Short (10 s) videos Quick videos Humorous/funny videos
	Viral challenges and campaigns	Ice bucket challenge Awareness challenges Viral campaign and viral songs
	Virtual reality gaming	Virtual reality at festival stalls Virtual reality for the general public Virtual reality video games in schools Virtual reality in clubs
	Mobile applications	Dating applications Educational applications
	Public figures	Celebrities Influencers Drag queens Sportspersons
Offline communication	Place‐based advertising	Murals at car parks Billboards near hospitals and sports clubs Public transport Bus stops and train stations Gay bars and clubs Gender clinics Toilets Gym and sauna Safety clips at an airline security instruction
	Broadcast media/traditional media	Infographic fatigue with social media Radio advertisements Television advertisements/shows Print media
	Merchandise	Campaigning using merchandise through various groups Wristbands/bands for people in college Novelty items Stress balls key chains Fidget balls with lumps Yoga mats
	Word of mouth	Word of mouth through local clubs/groups Award gala/fundraiser events Relatable guest speaker
	Games	Street games (Blind Box with dummy testicles) Party games
Behavioural targeting and education	Using the shock element	Massive writing with real figures and death statistics (shocking statistics) Shock factor and tactics to retain interest
	Overcoming stigma	Normalising taboo and stigma Overcoming stigma and embarrassment Conversation about body image
	Education	Creation of long‐term memory with associated story Including in school curriculum (health classes for boys)
Campaign frequency and reach	Same content on rotation	Same campaign on rotation Slogan on rotation Refreshing the campaign without compromising content
	Changing delivery approaches	Same core values and same information, but different imagery Starting on social media then moving to bus shelter
	Repeating the message	Repetitive messaging Building long‐term memory of the campaign
	Seasonal delivery	Festivals Pride
	Piggyback on another campaign	Cancer awareness Breast cancer awareness campaigns work
Demographic segmentation	Demographic segmentation by age	Secondary school boys College and university students Men 30–40 years of age Men at the highest risk Older generations and older men to break toxic masculinities
	Demographic segmentation by gender identity and sexual orientation	LGBT+ groups in different geographical areas Gay men and MSM Transgender and gender‐diverse people
	Inclusivity	Not limiting to one cohort of people (dangerous) Men of all shapes and sizes
	Larger demographics/communities	Labourers (e.g., farmers, construction workers) Local and national regions Mothers, family and social network
Campaign identity	Taglines and catchphrases	‘Test your Testes’ ‘Two to Tango’ ‘Have you checked your bales [of hay] today?’ ‘Do you know your nuts?’ ‘Know your nuts’ ‘Have you checked your balls?’ ‘Can you feel it?’
	Hashtags	#Knowyournuts #Checkyourself
	Music and songs	Musicals Choir
	Stickers and atlas	Upside down heart sticker Testicle atlas with 360 types of penises
	Advertisement with different shapes of balls	Sports advertisement to check the balls Advertisement with funny‐shaped balls (e.g., rugby balls) Christmas decorations/baubles ‘What's in your sack?’ Easter bunny with eggs
	Animation and cartoon characters	Squirrels with nuts Animated character

Abbreviations: LGBQT+, Lesbian, Gay, Bisexual, Transgender and Queer+; MSM, men who have sex with men.

### 3.2 Theme 1: Online communication

Using social media to deliver the campaign featured strongly in all three rounds of conversations, mainly because ‘pretty much everyone has a phone’ and ‘that's where absolutely everyone's on’. Facebook^TM^, Instagram^TM^, TikTok^TM^ and Twitter^TM^ (now X^TM^) were particularly mentioned. One participant suspected they had testicular cancer and sought help accordingly after seeing a Facebook^TM^ post:

I went [to the doctor] because I saw a friend's brother had a post on Facebook^TM^. And that kind of started ringing alarm bells in my head. I was like, oh, it's very similar to what I have … I better go and get checked. So, there is a value in having that kind of dialogue out there on social media that will trigger somebody. TikTok^TM^ and those sites will be valuable … I shared my own [story] on Facebook^TM^ just for that reason. Because I saw his [post], and I think it saved me.

Online videos were recommended. Many participants, however, warned against using long videos. Instead, they suggested creating ‘quick’, ‘funny, snappy, but informative’ videos to maintain people's attention:

It doesn't have to be a two‐minute video explaining everything. You can get it done in 10 seconds. And just convey those main messages in that 10 second … people look at something 10 seconds before they lose their attention. So, it's got to be 10 seconds.

Participants recalled challenges like the ice bucket challenge and suggested having similar viral social media challenges as part of the proposed campaign:

Maybe there's something we could do to put it [campaign] together, like maybe a little campaign, a song becomes something, and the soccer guys and the rugby guys and then the running guys make some sort of viral campaign … they could come up with a funny song that might go viral, something like that … maybe humorous to start off with. Getting the message across.

During the context‐setting presentation, the host discussed his experience leading research where virtual reality was used to promote young athletes' awareness of testicular diseases. Consequently, virtual reality gaming was recommended to spread the campaign messages and normalise testicular self‐checking, akin to breast self‐checking:

You can make a stall in a festival and use the VR [virtual reality] game that they have and show people … just let the message be.

The actual bit about the normal look and feel, this is why this is so difficult … feeling breasts is far more normalised than testicles, isn't it? so how do we normalize this? VR obviously is a very clever way of doing it.

Dating and educational mobile phone applications were also recommended to drive public engagement with the campaign:

Maybe design an app and grab the attention of young men. Possibly get a comedian to deliver the message on the app. Might be a fun way to do it. Might get their attention more than someone just being serious about it…

In keeping with having someone as the face of the campaign, participants recommended having celebrities, drag queens, influencers and/or sportspersons contribute to campaign delivery:

I would be matching a celebrity in the arts, maybe a drag queen, together with a very famous sport person. It doesn't have to be someone that has recovered from cancer. But, like, if someone has, you know, a story of having recovered from an injury or, like, building awareness towards health and how it looks like to be healthy, it could be good.

Others, however, suggested that the face of the campaign does not have to be a celebrity, referencing a memorable touristic campaign that they came across in two national airports which used ‘photographs of normal people’.

### 3.3 Theme 2: Offline communication

Participants warned against becoming ‘overly reliant on social media because it can cause infographic fatigue’. They recommended posters, newspaper articles and radio and television advertisements to scale up the campaign and reach out to individuals who do not use social media:

A radio show would reach a whole different community.

If you're going to go for something mass, like that would be together with TV and social media, I would say we need to go big.

We need to also think about utilising print media … things like posters, newspaper articles, it could be radio, because not everybody is online…

Place‐based advertising was proposed in all rounds of conversations. This involved delivering the campaign ‘where people are standing stationary for a few minutes’ and inadvertently ‘have no choice but to read’ and engage with the campaign. Some examples listed include: ‘mural in a car park’, ‘billboards’ near hospitals and gyms, ‘billboards inside a sauna’, ‘posters on trains and on bus’, ‘posters with QR [Quick Response] code that could be put in the bus stops where people can just scan and get more [information]’, ‘posters in sports clubs or gay bars’, ‘posters in gender clinics for transgender and gender diverse community’, posters in ‘STI [sexually transmitted infection] clinics’ and ‘posters in toilets, above urinals or on the backs of toilet doors’ and in ‘locker rooms’.

Merchandise such as wristbands, pins, pens and stickers bearing the campaign logo and slogan was also recommended for campaign delivery. Participants proposed using bespoke novelty items like ‘little testicles that are hanging from a bicycle’, ‘fidget balls … some have different lumps inside them’, ‘manufactured stress balls in the shape of testicles’ and ‘yoga mats’. Street games that can be used during parties were also proposed to drive engagement. The ‘Blind Box’ idea was introduced by one participant and was welcomed by others:

For me, the first question is, when you're feeling, what are you feeling for? As mentioned [in the host's context‐setting presentation], it's like you're looking for a knot in the field of knots. So, my idea would be a street game. For 18 and plus, it's a Blind Box. Inside you have two types of prosthetic testes. One is a, let's call it a healthy teste and the other one is an unhealthy teste. And the participants try to feel and figure out what is it that they're feeling, and the winner gets bragging rights. And it's a fun street game. It raises awareness. Everybody wants to try it and they're learning whilst they're trying it. And once you touch and feel something, you cannot un‐touch it.

Word of mouth was another strategy recommended for offline campaign delivery, either during public award ceremonies or via local LGBTQ+ clubs/groups. This was believed to bring people together and reach out to a wider community:

…The [name of LGBTQ+ running group] or the [name of LGBTQ+ rugby club] or the [name of LGBTQ+ soccer club] get them behind the campaign and then word of mouth, they'll pass that on kind of thing … basically talking to all four or five groups. Sort of creating a campaign, with each group doing something.

### 3.4 Theme 3: Behavioural targeting and education

Participants discussed the content of the campaign that they believed would drive engagement. Some thought that ‘the shock factor is needed to retain someone's interest and to spread the message’:

When we're in college we have posters put up for statistics on STIs and that, I just saw this one on, it's like massive writing and it's like, this is how many people between this age group gets this and it's like stuff that you wouldn't have thought of it until it's there in front of you and it kind of instils fear a little bit. It's like, oh Jesus! that could actually happen to me.

Others, however, cautioned against using fear tactics and recommended reverting to education instead:

So, people don't become overwhelmed, because the shock tactic doesn't work. If it's going to be all doom and gloom, people are just going to discount it. I don't want to know about it … like the ice bucket challenge is a shock, but it doesn't mean that you're scarred for life because of it … they could get more information from inside the resources of the organization.

Some participants seemed hesitant when discussing medical help‐seeking for testicular symptoms of concern. They expressed concerns around privacy, stigma and embarrassment:

I kind of wrote down about embarrassment and the stigma around it [testicular diseases]. Because this is just from my experience anyway and just from my own personal perspective … if I was to seek advice, how would I do it? And is there an anonymous way to do it? Because sometimes when you're looking at a health professional in the eyes, whether it's a male or a female, there's a level of embarrassment. So how do you remove that?

One participant speculated that the embarrassment has a cultural underpinning:

So, I've been in Ireland very [long] … there's an awful lot of shame around your body here in comparison to where I come from … to know your body and know what it's supposed to feel like and what normal should feel like, you should then be able to identify the normal and what is urgent….

As a result, the importance of normalising talking about one's testes and breaking taboo and stigma attached to testicular health was recommended as an integral part of the campaign:

It [campaign] shouldn't be comfortable. If it is comfortable, you are not catching anyone's attention. There is a lot of taboos around this. It really needs to be normalised. You can't normalise it without pushing boundaries.

Advertisements to overcome embarrassment were believed to push the boundaries and grab attention. The use of nudity and body positivity were particularly mentioned:

I was thinking about challenges like nudity overall is funny, right? It's funny, silly. It's also taboo. So, you know, when they do the naked cycle or like a naked run.

We don't do nudity very well. We are not good at showing our bodies. Maybe the advert could begin with that. Let's normalise.

### 3.5 Theme 4: Campaign frequency and reach

There was consensus that campaign delivery must be dynamic while, at the same time, ensuring that the messages are not diluted or lost. Participants cautioned against one‐off campaign delivery. Instead, they proposed repeating the campaign messages to build a long‐term memory:

It might be that it was last year I saw it [campaign], I forgot about it, and I'll see another campaign this year, and it might trigger [memory]. This idea of repetitive messaging is really important to create long‐term memory.

One participant recommended having several slogans ‘on rotation’:

…a number of short, snappy slogans and take the same campaign on rotation. So, if there was maybe four slogans … like short sentences with something like shocking or heavy or whatever, and then having the title slogan on rotation, but it's always the same message and you need the same content. So, you are kind of refreshing it without compromising on the content or your message.

Others proposed changing and rotating delivery approaches, while also stressing the importance of keeping to the same messages. A phased approach to campaign delivery was mentioned, starting with online then moving to offline delivery:

It [campaign] needs to have the same core values and the same information, but the image needs to change or the content of it would need to be updated. I think kind of starting it off maybe as a social media campaign for a few months and then changing it to a bus shelter campaign for a few months and then targeting or utilizing all the different approaches, but at varying times so people can see it in their various kinds of settings….

Seasonal campaigning was also recommended, where the campaign is customised for and delivered during various holidays and occasions like Christmas, Easter, Pride and certain festivals:

Maybe at particular times like festivals or … Pride, is that an easy one … like particular times where you have a specific, you have a large amount of specific audience maybe.

One participant suggested that the campaign ought to ‘piggyback on another campaign’ that is memorable and successful. Breast cancer campaigns were particularly mentioned:

I wonder, would you piggyback it [campaign] on another campaign? when I think about cancer awareness, I immediately think of breast cancer, but that's just me because I think that campaign has worked so well.

### 3.6 Theme 5: Demographic segmentation

The ‘one‐size‐fits‐all’ approach was perceived as ineffective in campaign design and delivery. Accordingly, participants emphasised the importance of being inclusive and tailoring the campaign to various age groups, gender identities, sexual orientations and ‘men of all shapes and sizes’. For instance, demographic segmentation by age was proposed, with differentiations made between secondary school boys, college and university students, men aged 30–40 years and older men:

Secondary school boys … a sense of shame and embarrassment there, and a lack of education. So, I think they would probably be a good group to target, particularly maybe in an all‐boys school.

Men 30‐40 to inform how to check yourself and where to seek help also to ensure that they understand median age [age group at risk of testicular cancer as per the host's context‐setting presentation] and that if caught early it is super treatable.

Target maybe older men … the toxic masculinity is so bad. Sometimes they [older men] can get a sign of weakness or they don't want to be a burden…

There was agreement that the LGBTQ+ community can be hard to reach since ‘not all gay men go to [name of a local gay bar], not all gay men are out and not all gay men are in same‐sex relationships’. Another challenge related to reaching out to transgender and gender‐diverse individuals:

We can't exclude the trans community because there are women who have testicles. It's important to maintain the health of those because that's an overall body Issue … it's quite a disparate experience to have testicles, if you identify as a woman. So, I think that it's something that could result in poor health seeking behavior. So, I think it's a grouping that does need to definitely have specific targeting … so, if you want to cater to some guy who goes to the gym all the time versus, like, every femme, trans woman. They're two very different demographics. So, how do you cater to both of them?

Participants believed that the campaign should also target labourers such as farmers and construction workers. They suggested leveraging pre‐existing platforms to reach out to larger groups locally and nationally:

You have groups that can spread the message even further. So, you're really hitting the local as well. You're really doing it on the ground, a big picture from the top. National, church, everywhere.

The role of the family in educating men about testicular diseases featured in some conversations. Participants believed that young girls are taught to check their breasts, while young boys are not educated about testicular self‐examination:

My mother told me how to wash my hair, how to brush my teeth, how to potty train … I remember my mum having conversations with my sister how to check her breasts for lumps … she never has that conversation with me which is interesting.

As a result, targeting female family members, particularly mothers and sisters, was recommended to spread the campaign message:

Targeting mothers and sisters? because you want to be able to have that conversation with your family to say, do you check yourself? And because it's kind of taboo, right? … so. Why limit yourself to just men?

### 3.7 Theme 6: Campaign identity

One of the WC tables was dedicated to building the campaign identity, namely, its logo, tagline/catchphrase and hashtag. Participants proposed using wordplay in advertisements. Light‐hearted words like ‘balls’, ‘nuts’, ‘eggs’, ‘bales [of hay]’ and ‘sack’ were recommended. Accordingly, slogans such as ‘Test your Testes’, ‘Two to Tango’, ‘Have you checked your bales [of hay] today?’, ‘Do you know your nuts?’, ‘Know your nuts’, ‘Have you checked your balls?’, ‘Judge a date by his nuts’, ‘Check your balls today!’, ‘Are you a nut expert?’, ‘Jingle Balls’, ‘What's in your sack?’ and ‘Can you feel it?’ were suggested, so were the hashtags #Knowyournuts and #Checkyourself. The use of humorous music and songs was also recommended:

A formally dressed choir, singing about testicles, I think would be really funny. You'd be throwing balls in front of them. Jingle balls. Jingle balls, jingle balls, balls … balls … balls …. Jingle balls, Jingle balls.

Other participants suggested an advertisement in a sports context:

An advert that has got somebody who's responsible for coaching a team that's bringing the balls to the training session or something. They open their boots [trunk] and they're not there. And everyone's there going…you didn't check your balls? Check your balls today!

Seasonal advertisements, particularly during Christmas and Easter were proposed, while also using wordplay:

If the campaign is a quick video ad, and you can actually very, very easily adjust that according to the time of year. So, you can have Santa searching for something in his big sack. So that's kind of funny. It can be PG [Parental Guidance] … and for Easter, bunny and his egg and again, it doesn't have to be overly explicit. It can be PG for both adults and for kids.

Some participants sketched what they thought would be the ideal campaign logo. The idea of the ‘upside down heart’ was presented by one participant:

You had a circular sticker, and you had a little drawing of testicles where, say, upside down, you could see a little love heart kind of a thing … When you take a love heart and put it upside down it kind of looks like testicles.

Another idea was to have an animated/cartoon character such as a squirrel as the campaign mascot:

You could have cartoon characters with nuts talking to each other and…even like a squirrel, you know the way they check them [nuts] … they like have them with their claws and they're checking them.

## 4 DISCUSSION

Recommendations on how best to deliver an inclusive community‐based ‘testicular awareness’ campaign are presented in the current study. We note this in the context that behaviour change in relation to testicular awareness is a product of the intersection of capability, motivation and opportunity, which can be spurred on by well‐designed, credible health campaigns.^36^

Participants recommended quick, funny, snappy and informative videos (i.e., short‐form videos) on social media. Currently, there are over 4.8 billion social media users. This number is projected to increase to more than 6 billion in 2027.^37^ Users spend on average 2.5 h on social media per day.^37^ The number of users is highest among men and individuals aged between 18 and 34 years.^37^ Short‐form videos on social media have been shown to command high engagement among online users.^37^ In a health context, social media is the fastest means to communicate health information either by sharing multimedia (e.g., text, images, audio and video) or leveraging various media forms (e.g., hyperlinks in posts on one platform can direct the public to other online resources).^38^ Over the past decade, the use of social media to access health information has increased significantly among the LGBTQ+ community.^39^ For these reasons, and in line with current study findings, social media platforms are recommended for campaign delivery.

The content and quality of health information on social media, however, are often questionable, particularly in the era of misinformation or ‘fake news’. A study evaluating the accuracy of information on genitourinary malignancies on Facebook^TM^, Twitter^TM^, Pinterest^TM^ and Reddit^TM^ found that the prevalence of inaccurate or misleading articles was high and that inaccurate articles were 28 times more likely to be shared than factual articles.^40^ Similarly, studies that assessed the content, reliability and quality of information regarding testicular cancer on YouTube^TM^ found that the quality of the content was poor.^41^, ^42^ Even when accurate information is shared, there is a risk that the campaign message is lost. In their analysis of tweets associated with the Movember campaign in Canada, Bravo and Hoffman‐Goetz^43^ found that only 0.6% of all tweets referenced prostate or testicular cancers, with most tweets focusing on community engagement activities and growing a moustache, rather than men's health. Likewise, a video of a female celebrity lying in bed while demonstrating testicular self‐examination using a red plum received 209,049 hits on YouTube over 27 months. Viewers' comments, however, were predominantly sexually explicit.^44^ This highlights the difficulty in designing a campaign that both triggers and maintains interest whilst emphasising the essence of health‐promoting messages.

To ensure wider reach, participants proposed offline campaigning using posters, billboards, merchandise, newspaper articles and radio and television advertisements. Offline communication, a conventional way of delivering health information, can reach out to a wider population, particularly individuals who do not use social media, as well as older men and men who have low literacy and/or health literacy levels.^45^ Traditionally, testicular cancer awareness campaigns have been delivered offline, with well‐documented evidence of effectiveness, albeit in the short‐term.^20^, ^22^ Engaging at‐risk younger groups with a testicular awareness campaign requires outside‐the‐box thinking. Men in the study by Saab et al.^46^ recommended moving away from print media. Instead, they recommended strategies that are innovative, light‐hearted, snappy, simple, brief and visually appealing to promote testicular awareness. A virtual reality game was developed accordingly. Its feasibility, usability and potential effectiveness in promoting testicular awareness have been established in previous research.^8^, ^27^ This highlights the potential to integrate virtual reality in the proposed campaign.

The sum of possible sources of information that an individual may encounter or consult incorporates both, information they actively seek out or passively encounter in their daily information sphere.^47^ Regardless of whether current study participants' preferences were for online or offline campaigning, their recommendations were clearly geared towards promoting testicular awareness passively, through intermediary channels rather than having the public actively seek the information. This is reflected in participants' suggestions relating to place‐based advertising in spaces ‘where people are standing stationary for a few minutes’ and inadvertently ‘have no choice but to read’ and engage with the campaign.

There was consensus that campaign delivery ought to be dynamic. Recommendations were made to deliver various slogans over a prolonged period, customise the campaign for various holidays and piggyback on other successful campaigns. Targeting female family members was also recommended to reach out to at‐risk groups indirectly. However, this might place the burden of care on women rather than empowering men to take ownership of their own health.^48^ It also may be problematic where women are not the preferred partners of men (e.g., gay men).

Participants proposed using humorous music and songs, advertisements in a sports context and seasonal advertisements to deliver the campaign messages. They were given creative license to design the campaign logo and slogan/catchphrase. Humorous words like ‘balls’, ‘nuts’, ‘eggs’, ‘bales [of hay]’ and ‘sack’ were used to designate the testes. The importance of accessible, male‐oriented, language in health interventions for men has been noted elsewhere.^48^ However, humour and puns should be used with caution to avoid offending individuals and to prevent the campaign messages from getting lost or diluted.^49^

Embarrassment due to the sensitive nature of testicular symptoms is a known barrier to medical help‐seeking.^17^ Some participants believed that the shock factor might help overcome such embarrassment. The risks and benefits of shock advertising or ‘shockvertising’ are widely debated, with some studies finding this type of advertising ineffective,^50^ and others reporting the public grows immune to ‘shockvertising’.^51^ Thus, its value to communicate information or consequences of inaction to motivate change may be limited.

Study participants emphasised the importance of being inclusive and tailoring the campaign messages and visuals to various age groups, gender identities, sexual orientations and occupations. As such, they recommended targeting various demographic segments differently. Indeed, tailoring interventions instead of adopting a ‘one‐size‐fits‐all’ approach is recommended in the development of effective health promotion interventions,^52^, ^53^ such as the campaign that the authors of the present paper set out to develop. The application of the intersectionality framework^54^ to inclusivity in this context asks us to consider that individuals are best understood through lenses that are sensitive to dimensionality, co‐occurrence and interlocking factors that are shaped by an individual's gender, race and culture. In addition, broader concepts that can render some groups to be alienated referred to within diversity marketing literature such as diversity in body types and physical abilities ought to be considered.^55^

## 5 LIMITATIONS

While various organisations and individuals were approached and invited to participate, 17 participants were included in this study and data were collected in a single site, limiting the transferability of findings. While nonprobability sampling was appropriate for the chosen methodology, this could have led to sampling bias. The presence of 17 individuals in one venue and the use of voice recorders could have led some participants to hold back and not discuss their thoughts and ideas fully. We attempted to build a hospitable place by turning the venue into a café‐like setting to address this risk. Indeed, many participants felt comfortable disclosing personal experiences as part of the process including personal experiences of a testicular cancer diagnosis.

## 6 CONCLUSION

Rich data were gleaned from WC workshop participants to co‐design and deliver an inclusive community‐based ‘testicular awareness’ campaign. Social media platforms are recommended to spread the campaign messages. Demographic segments that do not use social media ought to be targeted differently, for example, via radio, television and print advertising. The campaign must consider the embarrassment surrounding testicular diseases and signpost the public to sources of help and information. A multimodal, dynamic and phased campaign delivery is suggested, starting with online delivery to build the campaign profile then moving to offline advertising to ensure wider reach and better behaviour change in relation to testicular awareness. Light humour is an integral element of the campaign and the use of wordplay like ‘balls’ or ‘nuts’ may be effective. Partnerships built with representatives from key organisations ought to be leveraged to help facilitate campaign endorsement and ensure wider reach. Future research will be needed to evaluate the feasibility, acceptability, cost and effect of the campaign on promoting testicular awareness and early detection of testicular diseases.

## AUTHOR CONTRIBUTIONS

Mohamad M. Saab is the principal investigator. He was responsible for project conceptualisation, funding acquisition, project administration, data collection, data analysis and writing the original draft. Varsha N. Shetty contributed to data collection, data analysis and writing the original draft. Megan McCarthy, Martin P. Davoren, Angela Flynn and Josephine Hegarty contributed to project conceptualisation, data collection, data analysis and reviewing and editing of the manuscript. Ann Kirby, Steve Robertson, Gillian W. Shorter, David Murphy, Michael J. Rovito and Frances Shiely contributed to project conceptualisation and reviewing and editing of the manuscript.

## CONFLICT OF INTEREST STATEMENT

The authors declare no conflict of interest.

## ETHICS STATEMENT

This study received ethical approval from the Social Research Ethics Committee at University College Cork (Log 2022‐227). All participants were required to provide written informed consent before data collection.

## Data Availability

The data that support the findings of this study are available from the corresponding author upon reasonable request.
